# The Efficacy of Eye Movement Desensitization and Reprocessing Treatment for Depression: A Meta-Analysis and Meta-Regression of Randomized Controlled Trials

**DOI:** 10.3390/jcm13185633

**Published:** 2024-09-23

**Authors:** Ji-Woo Seok, Joong Il Kim

**Affiliations:** Digital Health Research Division, Korea Institute of Oriental Medicine, Daejeon 34054, Republic of Korea; suk6124@kiom.re.kr

**Keywords:** Eye Movement Desensitization and Reprocessing, depression, meta-analysis, meta-regression, psychotherapy, effectiveness

## Abstract

**Background**: Eye Movement Desensitization and Reprocessing (EMDR) therapy has gained attention for its potential effectiveness in treating depression beyond its initial use for PTSD. This systematic review and meta-analysis aims to evaluate the efficacy of EMDR in treating depression and to identify the variables influencing its effectiveness. **Methods**: A comprehensive search was conducted across databases, including MEDLINE, PubMed, and EMBASE, covering studies up to January 2023. A total of 521 studies were screened, and 25 studies with 1042 participants (522 EMDR, 520 control) met the inclusion criteria and were included in the meta-analysis. The inclusion criteria involved randomized controlled trials (RCTs) comparing EMDR to control conditions such as usual care or waitlist groups, with outcomes focused on changes in depression symptoms. **Results:** The results show that EMDR had a significant effect on reducing depression symptoms (Hedges’ g = 0.75), with moderate heterogeneity being observed. The meta-regression indicated that the severity of depression was a significant predictor of EMDR’s effectiveness, with greater effects in severe cases. Additionally, the systematic review analyzed and evaluated various theoretical models and related studies that explain how EMDR works for the treatment of depression, reporting on neurobiological models proposed in previous research. **Conclusions:** This study confirms that EMDR is effective in treating depression, particularly in severe cases, and highlights its potential as a non-pharmacological intervention. However, this study highlights the need for more standardized research and long-term evaluations to assess EMDR’s lasting impact. Integrating EMDR into multimodal treatment plans and primary care, especially for treatment-resistant depression, could significantly improve patient outcomes.

## 1. Background

Depression is a significant global mental health issue, with prevalence steadily increasing. Depression continues to be a growing concern, with mental distress significantly exacerbated by the COVID-19 pandemic. Depression rates in OECD countries saw an increase, with the prevalence of depressive symptoms remaining higher than pre-pandemic levels. While some countries like Korea, the United Kingdom, and the United States showed slight improvements in 2022, the prevalence remained at least 20% higher than pre-pandemic levels [1]. The OECD report also points to higher rates of depression among women and the elderly, exacerbating existing mental health disparities [1].

Depression significantly impairs individuals’ quality of life and functioning, and the World Health Organization has identified it as one of the leading causes of premature death and disability [2,3]. The socioeconomic cost of depression worldwide is immense, with estimates suggesting that it exceeds USD 1 trillion annually [4,5]. This figure underscores the critical importance of preventing and treating depression, not only to improve individual well-being but also to reduce the substantial societal and economic burdens [4,6]. Addressing depression through effective prevention and treatment strategies is essential for reducing these costs and improving overall public health outcomes [6].

Treatment for depression has significantly advanced in recent years, with the development of various options including pharmacotherapy, psychotherapy, and combinations of the two [7]. While pharmacotherapy is effective in alleviating depressive symptoms, it is reported that approximately 20–30% of all depression patients do not respond to medication [8]. Additionally, despite the introduction of cognitive behavioral therapy doubling the response rate, the relapse rate of depression after two years remains as high as 25% [9,10]. Given these limitations, there is an increasing demand for alternative treatment approaches, particularly for those who suffer from treatment-resistant depression. In this context, EMDR has emerged as a promising non-pharmacological intervention for depression. Originally developed for PTSD, EMDR has shown significant effectiveness in alleviating depressive symptoms, especially in severe cases. Moreover, the COVID-19 pandemic highlighted the adaptability of EMDR, as the shift to online therapy demonstrated its flexibility in providing continuous care when traditional face-to-face therapies were disrupted [11].

Previous studies have indicated that depression can be triggered and sustained by stressful life events and traumatic experiences [12,13]. Recent studies have reported that traumatic events, such as physical and emotional abuse, not only serve as significant psychosocial risk factors for major depressive disorder (MDD) but are also key contributors to the recurrence, persistence, and treatment resistance of depression [14]. Consequently, it has been hypothesized that Eye Movement Desensitization and Reprocessing (EMDR), a successful treatment for trauma, may also be effective in treating depression [15].

EMDR is a psychological intervention technique developed by Francine Shapiro in 1989 that is primarily used to treat symptoms related to traumatic memories [16,17]. This intervention aims to reprocess and integrate traumatic memories through eye movements, thereby reducing the negative emotions and somatic symptoms triggered by these memories. It includes an eight-phase protocol consisting of (1) history-taking and treatment planning, (2) preparation, (3) an assessment of traumatic memory, (4) desensitization, (5) the installation of positive belief, (6) a body scan, (7) closure, and (8) reevaluation [18,19].

Research indicates that EMDR taps into fundamental cognitive processes, such as attention, memory, and associative learning, all of which are critical in understanding and treating depression [20,21]. By guiding patients through bilateral stimulation and memory reprocessing, EMDR helps shift attentional focus away from negative, trauma-related information, improving emotional regulation and cognitive flexibility [19]. Additionally, EMDR may influence working memory by overloading cognitive resources, reducing the vividness and emotional intensity of distressing memories [22]. This process facilitates associative learning, allowing patients to form new, adaptive connections between past experiences and present emotions, which is crucial in restructuring maladaptive thoughts commonly found in depressive episodes [21,23]. Given that depression often involves dysfunctional cognitive patterns, such as rumination, memory biases, and impaired associative learning, the cognitive mechanisms engaged during EMDR play a key role in reducing depressive symptoms [23,24]. Understanding how EMDR interacts with these cognitive processes offers a deeper insight into its potential to treat depression, making it a valuable addition to the growing body of evidence supporting its broader therapeutic application.

Previous studies on EMDR therapy have reported that it may be effective in treating symptoms associated with major depression [25,26]. Ostacoli et al. (2018) conducted a study comparing EMDR and cognitive behavioral therapy (CBT) as adjunctive treatments to antidepressants in patients with recurrent depression. The results show that EMDR reduced depressive symptoms to the same extent as CBT both at the end of treatment and six months later [27]. Another study compared EMDR with trauma-focused CBT in patients with treatment-resistant depression, finding that while both therapies reduced depressive symptoms, EMDR had a greater effect, and only patients who received EMDR maintained continuous improvement in follow-up assessments [28]. A systematic review of EMDR studies applied to treat PTSD and pain reported that EMDR could significantly reduce not only PTSD symptoms but also co-occurring depressive symptoms [15,29].

Recently, a specialized EMDR treatment protocol for depression, known as DeprEND^®^, was introduced [18,30,31]. This protocol focuses on changing patterns of negative beliefs and self-blame associated with depression. According to previous studies, DeprEND^®^ has been reported to significantly reduce depression symptoms related to PTSD more effectively than CBT [32,33]. Despite the positive outcomes reported in EMDR-related studies to date, the generalizability of these results is constrained by the heterogeneity of the study designs and the variability in assessment methods employed.

A recent review of the effectiveness of EMDR therapy for major depression indicated that EMDR may be potentially effective for treating depression; however, it also highlighted methodological issues within the included studies [32,34]. The review specifically identified flaws such as a lack of randomization, small sample sizes, and a reliance on self-report measures, which could result in an overestimation of the intervention’s effectiveness. The authors concluded that additional research is needed to strengthen the evidence base [34].

Therefore, this study aims to update the evidence on the efficacy of EMDR by conducting a systematic review, including recent randomized controlled trials (RCTs). Additionally, a meta-analysis will be performed to provide a quantitative assessment of EMDR’s effectiveness in treating depression, thereby leading to more definitive conclusions. To further reduce heterogeneity among studies due to methodological differences, a meta-regression analysis will be conducted to explore specific variables that may contribute to heterogeneity systematically. By adjusting for the impact of these variables, this study aims to provide more reliable and valid evidence on the effectiveness of EMDR.

## 2. Methods

### 2.1. Study Design

This study involves a meta-analysis and meta-regression analysis conducted to evaluate the effectiveness of EMDR in treating depression.

### 2.2. Selection and Exclusion Criteria

This study was registered in PROSPERO for transparency (Registration ID: CRD42023401981) and was conducted in accordance with the Preferred Reporting Items for Systematic Reviews and Meta-Analyses (PRISMA) guidelines. Two reviewers independently screened the retrieved studies based on predefined selection criteria. The inclusion criteria for the meta-analysis were as follows: (1) Population: individuals diagnosed with depression according to depression assessment tools; (2) Intervention: EMDR therapy; (3) Comparison: control groups not receiving any intervention (e.g., no treatment, waitlist control, placebo, or usual care); (4) Outcomes: changes in depression symptoms; and (5) Study Design: randomized controlled trials and observational studies. The exclusion criteria were as follows: (1) studies involving patients currently on medication, (2) studies including other psychological therapies in addition to EMDR as an intervention, (3) studies that did not report depression outcomes, and (4) literature reviews, case reports, and qualitative studies.

### 2.3. Data Search and Selection Process

A literature search was conducted across electronic databases, including Google Scholar, MEDLINE, PubMed, the Cochrane Central Register of Controlled Trials (CENTRAL), EMBASE, Web of Science, PsycINFO, and ProQuest Dissertations, covering the period from the inception of each database to January 2023. The following key search terms were used: (depression OR depressive symptom OR Major depressive disorder) AND (EMDR OR Eye Movement Desensitization and Reprocessing OR eye movement psychotherapy) AND (randomized OR random OR randomly OR randomization OR RCT OR RCTs) AND (Waitlist OR TAU OR treat as usual OR no intervention OR CAU OR care as usual). Additionally, the reference lists of the identified studies and relevant articles suggested by meta-analyses and systematic reviews were manually reviewed. There were no restrictions on the country of publication, participant gender, or race.

After removing duplicate records from the literature collected through electronic databases and manual searches, the titles and abstracts were reviewed to initially select relevant studies. Full texts were then reviewed based on the inclusion and exclusion criteria to select the final studies. The selection process was independently conducted by two researchers. In cases of disagreement, the studies were re-evaluated based on the inclusion and exclusion criteria, and consensus was reached through discussion.

### 2.4. Quality Assessment of Included Studies

Two independent reviewers conducted a full-text review of the included studies and assessed the quality of each study included in the meta-analysis using the Cochrane Risk of Bias Tool for RCTs [35]. The quality assessment considered the following bias categories: selection bias (random sequence generation and allocation concealment), reporting bias (selective reporting), performance bias (blinding of participants and personnel), detection bias (blinding of outcome assessment), attrition bias (incomplete outcome data), and other sources of bias. Each of these domains was rated as having a low, high, or unclear risk. The quality assessment results were cross-checked, and in cases of disagreement between the reviewers, discrepancies were resolved through discussion or by consulting a third party to reach a consensus.

### 2.5. Data Extraction and Analysis

Based on relevant prior studies in the literature, a coding framework was developed, and two research assistants extracted relevant information according to this framework. The data extraction form included information on participant characteristics and intervention methods, such as title, author, publication year, participant age, gender, sample size, participant characteristics (e.g., refugees, PTSD, or phobias), type of control condition, intervention duration, duration per session, total number of sessions, depression measurement tools, and overall depression scores before and after the intervention. When post-intervention scores were reported at multiple follow-up points, only the assessment conducted immediately after the intervention was considered. The extracted data were cross-checked, and consensus was reached through discussion.

### 2.6. Statistical Analysis

Statistical analyses were conducted using the “meta” package in the R program (version 4.4.1). The summary effect size was calculated using a random-effects model while considering the variability in sample size, intervention methods, and duration across studies [36]. The effect size was assessed using Hedges’ g, which adjusts for the bias in Cohen’s d, especially when comparing the mean differences between two groups [37]. Cohen’s d tends to overestimate the effect size in small samples, making it difficult to accurately estimate the population’s standard deviation. Since many studies included in this meta-analysis had small sample sizes, the effect size was corrected using Hedges’ g [38,39]. An effect size smaller than 0.15 is interpreted as small, 0.40–0.74 as medium, and greater than 0.75 as large [40]. The effect size is interpreted with a 95% confidence interval, which indicates precision. If the confidence interval is greater than 0, the effect is considered significant; if it includes 0, it is not considered significant [39]. A narrower confidence interval indicates greater precision of the estimated effect size, meaning the estimate is closer to the actual effect [37].

In this study, heterogeneity among studies was visually assessed using a forest plot. Additionally, the Q statistic and I^2^ index were calculated. The Q value measures the variability among effect size estimates across studies, while the I^2^ index expresses the proportion of total variability attributable to heterogeneity as a percentage. Generally, a Q value with a significance level of 0.1 or lower or an I^2^ index of 50% or higher indicates substantial heterogeneity among the studies [41].

To explore the sources of heterogeneity observed in the meta-analysis and to provide additional explanations, a meta-regression analysis was conducted. This analysis assessed the moderating effects of study-level characteristics, such as the severity of depression, age, and total number of EMDR sessions [42].

## 3. Results

### 3.1. Selection of Studies

A total of 521 studies were identified through the literature search. Among these, 508 studies were retrieved from the database search, and an additional 13 studies were identified through the snowball sampling method.

**Figure 1:**
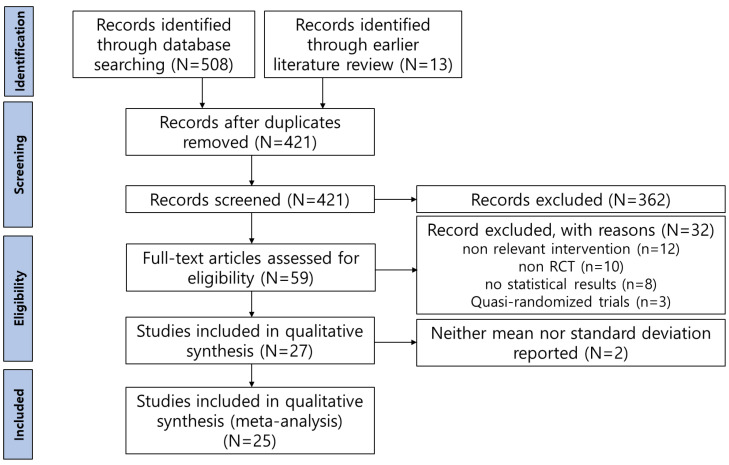
Flow diagram of study selection.

Initially, 421 duplicate studies were removed. The titles and abstracts of the remaining studies were then screened according to the inclusion and exclusion criteria, resulting in 59 studies being preliminarily selected. However, among these, 12 studies were excluded due to inappropriate interventions, such as comparing the effectiveness of EMDR with other interventions (ex., CBT and exposure therapy), using combined methods that included EMDR and the emotional freedom technique, or combining EMDR with pharmacotherapy. Additionally, 10 studies were excluded for not utilizing a randomized experimental design, 8 studies were excluded for not providing calculable statistical results, 3 studies were excluded for being quasi-experiments, and 2 studies were excluded because, despite providing effect sizes, it was not possible to extract the means and standard deviations for each group before and after the intervention. Ultimately, 25 studies were selected for inclusion in the final analysis (Figure 1).

### 3.2. The Characteristics of the Studies

The characteristics of the 25 studies included in the meta-analysis were summarized (Table 1). The summary includes information on the authors; year of publication; participant characteristics; age; number of participants; type of control group; duration, frequency, and period of the intervention; total number of intervention sessions; and depression diagnostic assessment.

The 25 studies analyzed were published between 1994 and 2023. Specifically, there were 2 studies from the 1990s, 6 studies published between 2000 and 2010, 13 studies published between 2011 and 2019, and 4 studies published after 2020. The participants included refugees, patients with PTSD, individuals with depression, patients with phobias, and those with bipolar disorder, all of whom met the criteria for a depression diagnosis based on the depression diagnostic assessment. Among the 25 studies, 3 focused on children and adolescents, while the remaining studies involved adult participants. The sample sizes in each study ranged from a minimum of 17 to a maximum of 83 participants, with a total of 522 participants in the EMDR intervention groups and 520 in the control groups. Regarding the control groups, 17 studies used a no-treatment control, 7 studies used usual care, and 1 study assessed both no-treatment and usual care controls.

The duration of EMDR sessions ranged from 50 to 90 min per session. In terms of the number of intervention sessions, 1 study conducted 1 intervention session, 12 studies involved 1–5 sessions, 9 studies had 6–10 sessions, and 3 studies included 12 sessions. Additionally, in four studies, the number of intervention sessions was not fixed but tailored to each participant’s need, and an average number of sessions was reported.

The most used assessment for measuring depression was the Beck Depression Inventory, which was utilized in 14 studies. Other tools included the Hamilton Depression Rating Scale, the depression subscale of the Hospital Anxiety and Depression Scale, the Children’s Depression Scale, the Patient Health Questionnaire, the Center for Epidemiologic Studies Depression Scale, and the Self-Rating Depression Scale.

### 3.3. Quality Assessment Results

We conducted a risk of bias analysis using Review Manager software version 5.4 (Nordic Cochrane Center, Copenhagen, Denmark). Table S1 presents a summary of the risk of bias for each study, indicating that 36% of the studies were assessed as having a low overall risk of bias. The main issues identified were the lack of an intention-to-treat analysis and insufficient reporting on allocation concealment.

While the majority of studies employed appropriate randomization methods, one study did not implement allocation concealment, and fourteen studies mentioned it but did not provide specific details. Of the 25 studies, 20 reported dropout rates, with the participant dropout rate ranging from 0% to 25%. Among the 16 studies with a dropout rate greater than 0%, 11 conducted an intention-to-treat analysis based on the group to which participants were originally assigned (Table S1).

### 3.4. An Analysis of the Effect Size and Moderator Effects of EMDR

**Figure 2:**
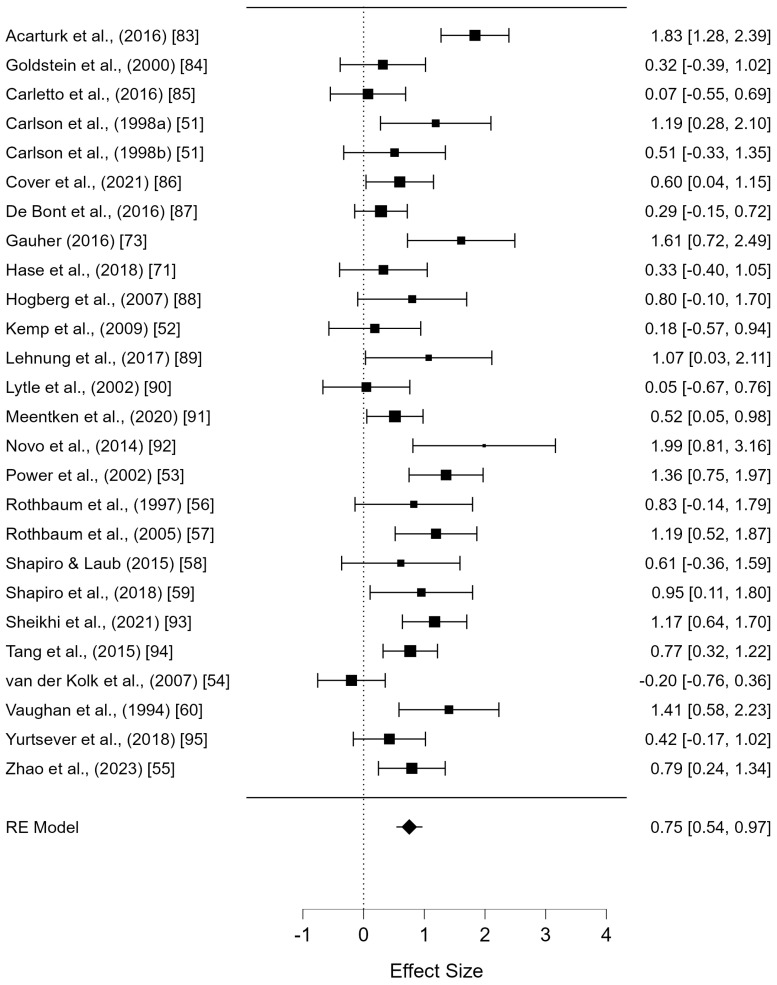
The effect of EMDR on depression [43,44,45,46,47,48,49,50,51,52,53,54,55,56,57,58,59,60,61,62,63,64,65,66,67].

The effect sizes (Hedges’ g) of the 25 selected studies (e.g., 26 trials) were calculated and presented in a forest plot (Figure 2). The overall average effect size was Hedges’ g = 0.75 (95% CI: 0.54–0.97), indicating a large and statistically significant effect. The heterogeneity of the studies, measured by the proportion of total variance attributed to between-study variance, was I^2^ = 62.80% (Q = 65.53, df = 25, and *p* < 0.001), indicating a moderate level of heterogeneity.

To explain the heterogeneity in effect sizes across studies, a meta-regression analysis was conducted using the sample size, number of intervention sessions, participants’ age (e.g., adults vs. children/adolescents), and participants’ depression severity (e.g., mild vs. severe) as moderator variables. When the sample size was used as a predictor, the estimate was −0.006, which was not statistically significant (*p* = 0.262). Similarly, the estimates for the number of sessions and participants’ age were 0.030 and 0.099, respectively, both of which were not statistically significant (*p* = 0.966 and *p* = 0.284). However, the estimate for participants’ depression severity was 0.602, which was statistically significant (*p* = 0.007). This indicates that the more severe the participants’ depression, the greater the effect size of EMDR (Table 2).

### 3.5. Subgroup Analyses

**Figure 3:**
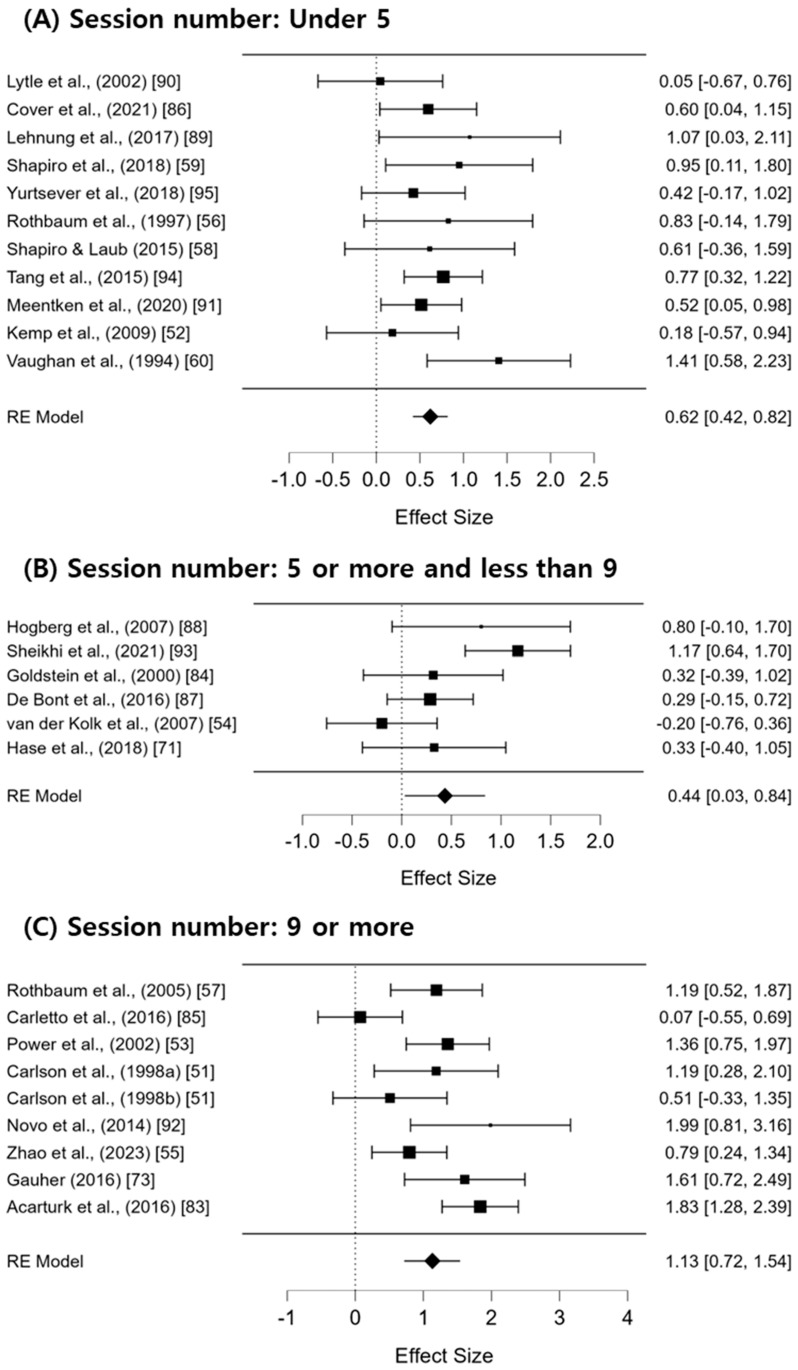
The results of the subgroup analysis based on the number of total sessions [43,44,45,46,47,48,49,50,51,52,53,54,55,56,57,58,59,60,61,62,63,64,65,66,67].

For the clinical application of EMDR, a subgroup analysis was conducted by categorizing the number of EMDR sessions into three groups: 5 or fewer sessions, 6–10 sessions, and more than 11 sessions. The results of this analysis are presented in Figure 3. The average effect size for each subgroup was statistically significant. Specifically, the effect size for 5 or fewer sessions was Hedges’ g = 0.62 (95% CI: 0.42–0.82; I^2^ = 0%; Q = 9.78; df = 10; *p* = 0.46); for 6–10 sessions, Hedges’ g = 0.44 (95% CI: 0.03–0.84; I^2^ = 62.56%; Q = 13.65; df = 5; *p* = 0.018); and for more than 11 sessions, Hedges’ g = 1.13 (95% CI: 0.72–1.54; I^2^ = 66.22%; Q = 24.54; df = 8; *p* = 0.002). The effect size was the largest for interventions with more than 11 sessions, though the heterogeneity remained moderate to high.

**Figure 4:**
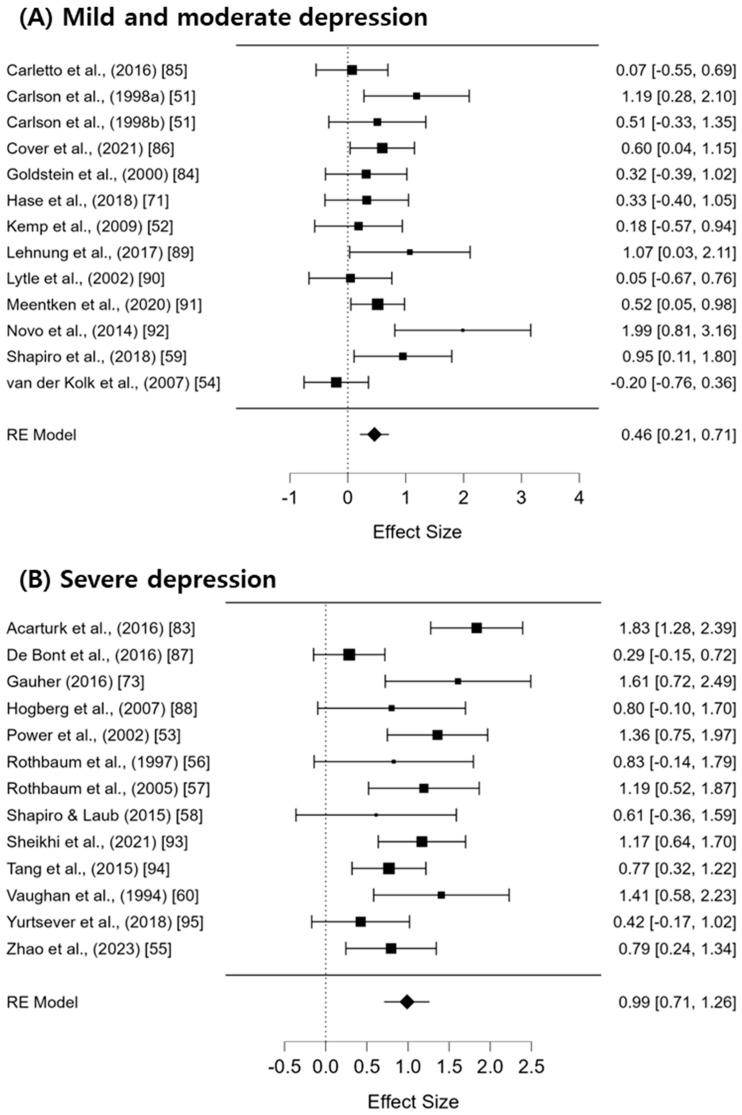
The results of the subgroup analysis based on depression level [43,44,45,46,47,48,49,50,51,52,53,54,55,56,57,58,59,60,61,62,63,64,65,66,67].

To examine the differences in EMDR effectiveness according to the severity of depression, a subgroup analysis was conducted by categorizing depression symptoms into mild and moderate-to-severe levels (Figure 4). For the group with mild depression, the effect size was Hedges’ g = 0.46 (95% CI: 0.21–0.71), indicating a moderate effect size with low heterogeneity (I^2^ = 35.85%; Q = 20.74; df = 12; *p* = 0.054). In the group with moderate-to-severe depression, the effect size was Hedges’ g = 0.99 (95% CI: 0.71–1.26), indicating a large effect size, with heterogeneity remaining moderate to high (I^2^ = 57.99%; Q = 29.36; df = 12; *p* < 0.05).

### 3.6. Analysis of Publication Bias

To verify the integrity and validity of the study results, a publication bias was estimated, starting with a funnel plot analysis to examine the asymmetry of effect sizes [39,68]. The funnel plot, which visualized the effect sizes and standard errors of the studies, showed that the data points were mostly symmetrically distributed around the mean effect size, although there are a few studies concentrated in the lower right corner (Figure S1). This suggests that publication bias is unlikely to have a significant impact on the overall results of the meta-analysis.

To objectively assess the asymmetry of the effect sizes, Egger’s regression test was performed. The result indicates that bias = 1.536 (*p* = 0.124), suggesting that publication bias was not statistically significant.

Given the slight clustering observed in the lower right corner of the funnel plot, the trim-and-fill method was applied to adjust for potential publication bias. After recalculating the corrected effect size, it was found that including one additional study would make the funnel plot symmetrical. The corrected effect size was adjusted from 0.75 to 0.73, confirming that the detected publication bias did not significantly impact the overall study results (Figure S2).

## 4. Discussion

The purpose of this systematic review, meta-analysis, and meta-regression is to update the latest research on EMDR intervention for depression, quantitatively analyze its effectiveness, and identify specific variables that influence the effectiveness of EMDR. The findings suggest that EMDR, which has been primarily associated with the treatment of PTSD, can also be effectively applied to treat mental health issues such as depression.

The result of the meta-analysis indicates that EMDR has a significant effect on treating depression, although a moderate level of heterogeneity was observed, and only eight studies were assessed as having a low risk of bias. The findings reveal that at the conclusion of the studies, EMDR therapy was more effective in treating depression compared to the control groups (e.g., usual care or waitlist) (Hedges’ g = 0.75, adjusted to 0.73 after trim-and-fill correction). The meta-regression analysis demonstrated that the effectiveness of EMDR was consistent regardless of the study methodology (e.g., number of sessions and sample size) or participants’ demographic characteristics (e.g., age), which aligns with previous research [25,69,70]. This suggests that EMDR therapy is consistently effective in reducing depressive symptoms across various conditions.

Additionally, the results of the meta-regression analysis indicate that the severity of depression is a significant predictor of the effectiveness of EMDR (z = 2.688; *p* = 0.007). The subgroup meta-analysis revealed that for mild to moderate depression, the effect size of EMDR was 0.46 (95% CI: 0.21–0.71), indicating a moderate effect. In contrast, for severe depression, the effect size was larger at 0.99 (95% CI: 0.71–1.26). This suggests that the more severe the depression, the greater the therapeutic effect of EMDR.

EMDR might be more effective for severe depression due to several neurobiological mechanisms. First, it might promote neuroplasticity, allowing for the reprocessing of traumatic memories, which could help reduce deeply entrenched negative beliefs and rumination [71,72]. Second, it might downregulate hyperactivity in the amygdala, potentially reducing emotional distress and stabilizing emotions [71,72,73]. Third, EMDR might target cognitive distortions and negative self-referential thoughts, leading to quicker cognitive shifts. It might also regulate the autonomic nervous system, potentially addressing physiological symptoms like insomnia and chronic stress [74]. Additionally, it might reduce avoidance behaviors, enabling patients to confront underlying trauma, and enhance prefrontal cortex engagement, which could improve emotional regulation [75]. These combined factors suggest that EMDR might be particularly effective in severe depression, especially when trauma is involved.

### 4.1. The Effect of EMDR on Depression Comorbid with PTSD

Previous studies on PTSD have reported that EMDR interventions improve comorbid depression [46,52,57,64,67]. When treating PTSD with EMDR, comorbid depression showed significant improvement. EMDR has been shown to be more effective than a waitlist control [46,52,57,58,59,60,61,65,67], no treatment [64], and even more effective than pharmacotherapy with antidepressants (e.g., fluoxetine) [64]. Additionally, EMDR has been reported to have effects similar to exposure therapy [59,76,77,78]. Other studies have also found that when compared to cognitive behavioral therapy (CBT), EMDR has a similar effect on improving comorbid depression, with no significant differences between the two interventions [57,79,80].

While these studies demonstrate the effectiveness of EMDR in the short term, there is increasing recognition of the importance of long-term outcome studies to fully understand the lasting impact of EMDR, especially in the context of depression. One study, which assessed the long-term effects of EMDR on adult female survivors of childhood sexual abuse, provides key evidence in this regard [81]. The results show that the benefits of EMDR on depression and trauma-related symptoms were maintained 18 months after treatment, with participants continuing to demonstrate significant improvements in Beck Depression Inventory scores. This suggests that EMDR’s therapeutic effects are not only immediate but also sustained over the long term, offering a more robust solution for trauma-related depression [81]. These long-term results underscore the importance of conducting further research into how EMDR can continue to provide lasting benefits for depression, especially for individuals with trauma histories. This is crucial for expanding the clinical application of EMDR and refining treatment protocols to maximize long-term efficacy.

### 4.2. The Effectiveness of EMDR on Major Depressive Disorder

EMDR has been reported to be effective in treating major depressive disorder (MDD) even in the absence of comorbid PTSD. A case study involving two adolescents with mild to moderate depression found that EMDR significantly reduced depressive symptoms, with remission being maintained for 2–3 months post-treatment [82]. Another case study reported the successful recovery of a patient with severe depression after three months of EMDR therapy [83], and a patient with depression comorbid with ADHD experienced such significant improvement that they were able to discontinue medication following treatment [84]. Additionally, a woman with chronic depression that was resistant to antidepressants reported complete remission of depression after nine sessions of EMDR, with remission maintained for six months [85].

RCT studies have also demonstrated the effectiveness of EMDR in treating MDD [27,49,50,86,87,88]. A large-scale RCT conducted across six European countries involving 30 patients with recurrent depression found that the group receiving combined EMDR and standard treatment showed greater improvement in depression compared to the group receiving standard treatment alone [50]. Gauhar et al. (2016) found that 26 participants diagnosed with MDD showed significant improvement in depressive symptoms after 6–8 sessions of EMDR, along with a reduction in negative cognitions. These improvements were maintained at a three-month follow-up, suggesting that EMDR can be an effective long-term treatment for depression [49].

Combining EMDR with other treatments for depression can significantly enhance treatment outcomes. Hofmann et al. (2014) reported that when EMDR is combined with CBT, it results in higher remission rates and greater reductions in depressive symptoms compared to CBT alone [31]. This suggests that EMDR may be particularly effective in addressing trauma-related components of unipolar depression. Similarly, in the European Depression EMDR Network Randomized Controlled Trial (EDEN), Ostacoli et al. (2018) found that EMDR, when used as an adjunct to antidepressant medication, led to slightly better outcomes in reducing depression symptoms compared to the combination of CBT and antidepressant medication, particularly in patients with recurrent depression [27]. Hase et al. (2018) also found that EMDR provided better outcomes when compared with medication alone, especially in treatment-resistant depression, by improving emotional regulation and processing unresolved trauma [50].

The rationale behind combining EMDR with other therapies lies in its ability to address trauma-related elements of depression that may not be fully resolved by cognitive restructuring in CBT or pharmacotherapy alone. EMDR complements these treatments by reprocessing unresolved traumatic memories, helping to reduce emotional distress and improve overall treatment outcomes. This multimodal approach suggests that EMDR can be a valuable adjunctive therapy, especially for treatment-resistant or trauma-related depression. Further research is needed to establish the most effective combinations and optimize treatment protocols for different types of depression.

### 4.3. Mechanism of EMDR in Treating Depression

The Adaptive Information Processing (AIP) model explains the therapeutic effects of EMDR on depression [19]. Based on the AIP model, if negative experiences related to stressful events are not adequately processed, they can become “frozen” in the brain, retaining the original emotions, thoughts, and sensations. These improperly processed memories can be triggered by internal or external stimuli, leading to distorted thoughts or emotions, and potentially contributing to mental disorders such as depression [19]. Barry et al. (2006) suggested that depression is closely related to a memory bias within the implicit memory system, where negative self-relevant information is more accessible than positive information [89]. This bias reinforces a negative self-concept and contributes to the persistence and worsening of depressive symptoms. Dysfunctional memories, resulting from incomplete processing, lack “memory awareness”, meaning that the emotional aspects of these memories are not properly integrated [89].

EMDR therapy helps reprocess these dysfunctional memories by using eye movements or other bilateral stimulation. This process influences the brain’s neural networks, allowing the dysfunctional memories to be integrated into existing semantic connections [90,91]. Specifically, EMDR activates the brain’s information processing system through bilateral stimulation, helping to properly integrate repressed or incompletely processed memories. This reduces the emotional burden of the memories, modifies the negative self-concept, and alleviates depressive symptoms [90]. EMDR also provides a safe environment for patients to express and process repressed or unprocessed emotions, which can be particularly beneficial for those with depression. As these repressed emotions are resolved, depressive symptoms may be alleviated [90].

Some researchers suggested that EMDR facilitates the reprocessing and integration of traumatic memories by inducing brain states similar to those experienced during specific sleep stages (i.e., Rapid Eye Movement, REM) [92,93]. During REM sleep, the brain reactivates memories, reduces their emotional intensity, and integrates them into broader semantic memory networks. EMDR is believed to induce a brain state similar to REM sleep, where memories are reactivated, desensitized, and integrated into broader memory networks, thereby reducing the emotional burden of traumatic memories and promoting psychological healing [92]. Stickgold et al. (2002) suggested that EMDR mimics REM sleep [92], while Pagani and Carletto (2017) proposed that EMDR might also induce a state similar to slow-wave sleep (SWS), which is crucial for memory consolidation and emotional processing [93]. Both theories highlight EMDR’s ability to leverage natural sleep-related mechanisms to reduce the emotional impact of traumatic memories and promote psychological healing.

### 4.4. Neural Mechanisms of EMDR in Treating Depression

The neural mechanisms of EMDR play a crucial role not only in treating PTSD but also in addressing depression. In particular, the key brain regions activated by EMDR overlap with those involved in depression. During EMDR therapy, bilateral alternating stimulation alters the neural networks activated during memory reprocessing, enhancing the function of the prefrontal cortex and anterior cingulate cortex [71,73]. These regions are critical for emotional regulation and decision making, and their function is often impaired in depression. Notably, EMDR increases blood flow in these areas, which is directly linked to the alleviation of depressive symptoms [71,94,95]. Furthermore, EMDR stimulates these brain regions through bilateral stimulation, enabling the reprocessing of traumatic memories stored in dysfunctional neural networks. This reduces the intensity of negative memories that contribute to depressive symptoms and promotes neuroplasticity, allowing the brain to form new, adaptive neural connections [71,73]. Additionally, EMDR influences theta cordance, a neural marker associated with depression, showing significant reductions after treatment, which correlates with improvements in depressive symptoms [95]. This suggests that EMDR plays a key role in modulating the imbalanced cognitive and emotional processes seen in depression.

The neuroplasticity promoted by EMDR decreases emotional reactions linked to past negative memories and facilitates the formation of more adaptive memories. In this process, long-term potentiation and long-term depression mechanisms play a crucial role [71]. These mechanisms help patients with depression move away from negative emotional responses and develop more realistic and positive neural connections. In conclusion, EMDR is effective in treating depression because these neural mechanisms enhance emotional regulation and memory reprocessing. Through these processes, EMDR can reduce the negative thoughts and emotional distress that characterize depression, potentially contributing to long-term psychological stability.

### 4.5. Limitations

While the results suggest that EMDR may be effective in treating depression, there are several limitations to consider. One limitation of the selected studies is the inconsistency in depression diagnosis across the studies. Out of 25 studies, only 5 explicitly diagnosed participants with depression, while the rest assessed subclinical depressive symptoms. This variation may affect the interpretation of the results, as subclinical symptoms differ from clinical depression in terms of severity and treatment. Additionally, the lack of detailed information on participants’ histories of depression, such as the age of onset, the number of major depressive episodes, and distinctions between MDD and bipolar disorder, limits the depth of analysis. If such data were available, they would allow for a more nuanced understanding of EMDR’s effectiveness across different depressive profiles, providing clearer insights into its impact on specific subgroups of patients. Future studies should ensure more consistent diagnostic criteria and collect detailed depression history to better assess EMDR’s impact on clinically diagnosed depression.

Another limitation is that many of the studies included in this meta-analysis had small sample sizes, which may overestimate treatment effects and limit generalizability, despite meta-regression confirming that sample size did not significantly affect EMDR’s efficacy. Additionally, potential biases in the methodologies of the selected studies, such as incomplete randomization and a lack of double blinding, may compromise the reliability of the results.

Furthermore, this meta-analysis only assessed the immediate effects of EMDR, even though a few of the included studies reported long-term outcomes. However, the follow-up periods across these studies varied significantly in both length and timing. This variability made it impossible to combine the long-term results into a cohesive meta-analysis. As a result, this analysis could not determine how effective EMDR is over an extended period. This limitation highlights the need for future research with more standardized follow-up periods to better assess the sustained impact of EMDR on depression.

### 4.6. Recommendations

In light of the research on the use of EFT for depression, several recommendations can be made. First, further investigation into the neural mechanisms underlying EFT’s impact on depression is crucial. The current findings show that EFT may alter brain connectivity in areas related to emotional regulation and mood, but more studies are needed to understand how EFT specifically affects brain circuits associated with depressive symptoms. Additionally, given the positive outcomes in treating depression, EFT should be integrated into broader, multimodal treatment plans. As a non-pharmacological intervention, EFT could complement existing therapies like CBT or medication, particularly for treatment-resistant depression. Moreover, while EFT has been well documented for psychological conditions like anxiety and PTSD, more research is necessary to evaluate its efficacy in treating physiological conditions that are often comorbid with depression, such as heart disease and cognitive impairments. Finally, EFT’s inclusion in primary care settings is recommended. As a safe, fast, and effective method for treating depression, it can offer long-term symptom relief with minimal side effects. Its integration into mainstream healthcare would provide an additional tool for managing depressive disorders, especially for patients who prefer non-drug treatments. These recommendations will help broaden the understanding and use of EFT in treating depression.

### 4.7. Conclusions

This systematic review and meta-analysis highlights the effectiveness of EMDR in treating depression, expanding its application beyond PTSD. The findings indicate that EMDR is consistently effective in reducing depressive symptoms, with greater effects observed in individuals with severe depression. The meta-regression analysis confirmed that variables such as the number of sessions and participant demographics do not significantly impact the therapy’s effectiveness, suggesting that EMDR can be broadly applied across different populations.

While this study reaffirms EMDR’s utility in managing both comorbid PTSD and stand-alone depressive disorders, limitations such as small sample sizes and inconsistent follow-up periods emphasize the need for more robust, standardized research. The analysis of neural mechanisms provides insights into how EMDR may influence brain functions related to emotional regulation and memory processing, offering a scientific basis for its therapeutic effects on depression. Future research should focus on long-term outcomes and more standardized study designs to assess the sustained impact of EMDR on depression. The inclusion of EMDR in multimodal treatment approaches and primary care settings, especially as a non-pharmacological option, can enhance treatment outcomes for patients with treatment-resistant depression. These steps will contribute to a broader understanding and utilization of EMDR as a powerful therapeutic tool for depression.

## Figures and Tables

**Table 1:** The study characteristics of the 25 studies selected for the meta-analysis.

Author and Year	Subjects	Depression Diagnosis	Age	Groups	Duration of Intervention	Session Number	Depression Assessment
Acarturk et al. (2016) [43]	Syrian refugees	no clinical diagnosis	T: 33.32 (11.09) C: 34.04 (10.00)	T: EMDR (*n* = 37), C: Waitlist (*n* = 33)	2 days/week, 4 weeks	8	Beck Depression Inventory
Goldstein et al. (2000) [44]	Patient with agoraphobia	no clinical diagnosis	22–63	T: EMDR (*n* = 18), C: Waitlist (*n* = 14)	1 day/week, 90 min/session, 6 weeks	6	Beck Depression Inventory
Carletto et al. (2016) [45]	Patients with multiple sclerosis	no clinical diagnosis	T: 39.52 (11.68) C: 40.66 (10.03)	T: EMDR (*n* = 20), C: TAU (*n* = 20)	1 day/week, 60 min/session, 12–15 weeks	10	Depression in Hospital Anxiety and Depression Scale
Carlson et al. (1998) [46]	Patients with PTSD	no clinical diagnosis	T: 52.7 (8.6) C1: 45.4 (3.5) C2: 46.9 (4.0)	T: EMDR (*n* = 10), C1: Waitlist (*n* = 12), C2: TAU (*n* = 13)	2 days/week, 6 weeks	12	Beck Depression Inventory
Cover et al. (2021) [47]	Patients with PTSD	no clinical diagnosis	T: 25.52 (7.93) C: 25.88 (8.23)	T: EMDR (*n* = 27), C: Waitlist (*n* = 25)	2 days/week, 105 min/session, 1 week	2	Depression in Hospital Anxiety and Depression Scale
De Bont et al. (2016) [48]	Patients with chronic psychotic disorders	no clinical diagnosis	T: 40.4 (11.3) C: 40.3 (9.7)	T: EMDR (*n* = 55), C: Waitlist (*n* = 47)	1 days/week, 90 min/session, 8 weeks	8	Beck Depression Inventory
Gauhar (2016) [49]	Patients with depression	major depressive disorder	18–60	T: EMDR (*n* = 13), C: Waitlist (*n* = 13)	1 day/week, 60 min/session, 6–8 weeks	7.3 (0.9)	Beck Depression Inventory
Hase et al. (2018) [50]	Patients with depression	major depressive disorder	T: 40.32 (9.25) C: 39.23 (10.02)	T: EMDR (*n* = 14), C: TAU (*n* = 16)	1 day/week, 90 min/session, 4–12 weeks	8.5 (2.41)	Beck Depression Inventory
Hogberg et al. (2007) [51]	Public transportation workers	no clinical diagnosis	T: 43 (8) C: 43 (11)	T: EMDR (*n* = 12), C: Waitlist (*n* = 9)	1 day/week, 90 min/session, 5 weeks	5	Hamilton Depression Rating Scale
Kemp et al. (2009) [52]	Adolescents with PTSD	no clinical diagnosis	6–12	T: EMDR (*n* = 13), C: Waitlist (*n* = 14)	1 day/week, 60 min/session, 4 weeks	4	Children’s Depression Scale
Lehnung et al. (2017) [53]	Refugees	no clinical diagnosis	32.4 (5.6)	T: EMDR (*n* = 12), C: Waitlist (*n* = 6)	2 days/week, 120 min/session, 1 week	2	Beck Depression Inventory
Lytle et al. (2002) [54]	Students with identified past stressful life experience	no clinical diagnosis	>18	T: EMDR (*n* = 15), C: TAU (*n* = 15)	1 day/week, 60 min/session, 1 week	1	Beck Depression Inventory
Meentken et al. (2020) [55]	Children with subthreshold PTSD	no clinical diagnosis	T: 9.8 (2.7) C: 9.4 (3.1)	T: EMDR (*n* = 37), C: TAU (*n* = 37)	1 day/week, 50 min/session, 2–5 weeks	3.5 (1.9)	Children’s Depression Scale
Novo et al. (2014) [56]	Patients with bipolar disorder	subsyndromal mood symptoms	T: 43.90 (6.87) C: 44.80 (6.86)	T: EMDR (*n* = 10), C: TAU (*n* = 7)	1 day/week, 90 min/session, 12 weeks	12	Beck Depression Inventory
Power et al. (2002) [57]	Patients with PTSD	no clinical diagnosis	T: 38.6 (11.8) C: 36.5 (11.6)	T: EMDR (*n* = 27), C: Waitlist (*n* = 24)	1 day/week, 90 min/session, 10 weeks	10	Depression in Hospital Anxiety and Depression Scale
Rothbaum et al. (1997) [58]	Sexual assault victims	no clinical diagnosis	T: 31.6 (9.8) C: 27.5 (11.1)	T: EMDR (*n* = 10), C: Waitlist (*n* = 8)	1 day/week, 90 min/session, 4 weeks	3	Hamilton Depression Rating Scale
Rothbaum et al. (2005) [59]	Sexual assault victims	no clinical diagnosis	33.8 (11.0)	T: EMDR (*n* = 10), C: Waitlist (*n* = 8)	2 days/week, 90 min/session, 9 weeks	9	Beck Depression Inventory
Shapiro and Laub (2015) [60]	Community critical incident	no clinical diagnosis	>18	T: EMDR (*n* = 8), C: Waitlist (*n* = 9)	2 days/week, 90 min/session, 1 week	2	Patient Health Questionnaire (PHQ-9)
Shapiro et al. (2018) [61]	People exposed to intensive rocket attacks	no clinical diagnosis	T: 41.7 (12.6) C: 36.2 (9.5)	T: EMDR (*n* = 12), C: Waitlist (*n* = 12)	3 days/week, 90 min/session, 1 week	3	Patient Health Questionnaire (PHQ-9)
Sheikhi et al. (2021) [62]	Patients with spinal cord injury	no clinical diagnosis	T: 34.6 (10.8) C: 38.5 (13.2)	T: EMDR (*n* = 32), C: TAU (*n* = 32)	1 day/week, 90 min/session, 5 weeks	5	Beck Depression Inventory
Tang et al. (2015) [63]	Adolescents who experienced typhoon	major depressive disorder	T: 14.24 (0.99) C: 14.48 (0.92)	T: EMDR (*n* = 41), C: TAU (*n* = 41)	1 day/week, 30–40 min/session, 4 weeks	3	Center for Epidemiologic Studies Depression Scale
van der Kolk et al. (2007) [64]	Patient with PTSD	no clinical diagnosis	T: 38.7 (14.3) C: 35.7 (13.4)	T: EMDR (*n* = 24), C: No intervention (*n* = 26)	1 day/week, 90 min/session, 8 weeks	8	Beck Depression Inventory
Vaughan et al. (1994) [65]	Patient with PTSD	17% of participants with major depressive disorder	20–78	T: EMDR (*n* = 12), C: Waitlist (*n* = 17)	1 day/week, 50 min/session, 3–5 weeks	4.3 (0.7)	Hamilton Depression Rating Scale
Yurtsever et al. (2018) [66]	Syrian refugees	no clinical diagnosis	T: 37.45 (11.08) C: 39.89 (10.96)	T: EMDR (*n* = 18), C: Waitlist (*n* = 29)	3 days/week, 1 week	2	Beck Depression Inventory
Zhao et al. (2023) [67]	Patient with PTSD	no clinical diagnosis	T: 25.5 (4.3) C: 24.6 (3.9)	T: EMDR (*n* = 26), C: Waitlist (*n* = 29)	1 day/week, 90 min/session, 12 weeks	12	Self-rating Depression Scale

Abbreviations: C, Control; PTSD, Post-Traumatic Stress Disorder; T, Treatment; TAU, Treatment as Usual.

**Table 2:** Meta-regression analysis of studies on depression symptoms.

Predictor Variables	Estimated Value	Standard Error	Z	*p*
Age	0.099	0.348	0.284	0.777
Sample size	−0.006	0.006	−1.123	0.262
Session number	0.030	0.031	0.966	0.334
Depression level	0.602	0.224	2.688	0.007

## Data Availability

The data supporting the findings of this study are available from the corresponding author upon reasonable request.

## Supplementary Materials

The following supporting information can be downloaded at: https://www.mdpi.com/article/10.3390/jcm13185633/s1, Table S1. Risk of bias. Figure S1. The result of publication bias. Figure S2. The results of the trim and fill analysis. The Prisma checklist [96].
